# Missed Labor

**Published:** 1867-10

**Authors:** T. B. Camden

**Affiliations:** Weston, West Virginia


"*Missed Labor.*" By T. B. Camden, M. D., of Weston, West Virginia.

This term was introduced by Dr. Oldham, he restricted it to cases "in which the full term of utero gestation has gone by without labor pains having set in, or the expulsion of the child affected."

As such cases are very rare, and the number recorded few. I have had my attention more particularly called to them from the fact that I had the misfortune to have one. And at the time had never remembered reading or hearing of a case of the kind, nor have I since, except the cases spoken of below.

The first of these cases is related in Guy's Hospital reports, by Dr. Oldham, under the caption of a " Rare case of Midwifery." The narrative states that the full period of utero-gestation was completed in June, and the woman carried the child until October, three months beyond the natural period. The function of lactation was established as soon as the usual period of gestation had expired. (She had lost a quart of blood in June.) The Dr. found the os uteri dilated sufficiently to introduce two fingers; but absolutely incapable of further dilation, and he believes the uterus would have given way before a greater degree of dilatation could have been effected. The usual excitants were given, ergot, electricity, ect., but without avail. The Dr. removed the arm and some other portions of the child, together with the placenta and cord through the os uteri. The uterus soon after diminished in size, the Dr. predicted the escape of the contents into the abdomen by ulceration. The post-moretm confirmed his opinion. The anterior walls were found to be removed by ulceration. The soft parts of the child had been taken away by the absorbents, and little else was left but the bones,

The second case was recorded in the April number of the *American Journal of Medical Sciences*, in 1853, and is related in a letter to Prof. Meigs by Dr. Hortze. This woman, I believe, carried her child near three years. A post-mortem put the matter beyond cavil. As I have not the Journal at hand, I cannot give the particular symptoms, ect.

The third case is by Dr. Wm. Johnson, of White House, M. J., February, 1855. The woman, aged 36 years, became enciente for the first time in the spring of 1852. Nothing of note occurred until December, when nine months of utero-gastation was completed. She had now pains, such as to induce her husband to call in her physician, he was called on in the evening and remained all night. The pains, however, were not severe, and wore off, *and never returned.* As in extra-uterine pregnacy, lactation was also here established at the prescribed period when utero-gestation should have been completed. She lived after this eleven months. Her health, however, soon began to give way after the abortive effort of the womb to expel the child. Dr. Johnson saw her for the first time in March. She had several turns of flooding; but the quantity of blood was not large. At this visit he found the os uteri obliterated, and the parietes of the under part of the womb thin. The tip of the finger entered the womb, and with it he touched what he thought the fœtal head. He says he employed considerable manipulation in order to increase the dilatation of the os uteri; but completely failed in the attempt. He says he might as well have attempted the dilatation of a piece of sole leather; to which alone he could compare it. It gave the same unyielding sensation. In consultation with Dr. Honeyman it was agreed to attempt the dilatation with sponge tents; secale cornatum was given for several days, all without effect, she gradually sank until fall, having lived eleven months. The post-mortum revealed a child within the womb; decomposition of the child had progressed, and the cranial bones were readily separable. The uterus fully embraced the child on every part of its surface. The head presented in the most favorable position for expulsion.

The fourth is by Dr. Green, of Cambridge, Ohio, and recorded in the "*Counselor*, June 30th, 1855," Columbus, O.—He was called on the night of the 25th of Feb. 1836, to see Mrs. T., aged about 43 years, in her sixth accouchment; found her to all appearance in labor. Shortly after his arrival she vomited freely, after which her pains left her. She rested well through the night and expressed herself as well as ever,

He says: "I left her expecting to be sent for during the night, but heard nothing from her for eight or ten days, when, passing the house, called to see her, when she informed me that on the day I left, the waters came away, and she had had a slight hemorrhage, which continued up to that time. That she had not felt the child move since her pains subsided, and she had a copious secretion of milk. I was satisfied that the child was dead, and gave as my opinion that labor would certainly take place in a short time and expel the child. But in this I was sadly mistaken, as in a few days afterward I was requested by her husband to visit her again. I found the discharge increasing, very offensive, and her health giving way. On examination I found the os uteri about the size of a silver dollar, and as *hard and as unyielding as an ivory ring.* I tried to force it to yield, but without success. I used ergot and friction to bring on contraction if possible, hoping that by so doing, with manipulation, I could force my hand through the ring, and remove the child, which I could easily feel; but the ergot had no effect. I stated the case to my patient and her husband, and gave them my opinion that the uterus could never be emptied by any other means than a division of this ring with the knife; to this proposition they would not consent. I tried again to force the os uteri to yield, with no better success.—In fact I might as well have tried to dilate an iron ring. I then advised consultation, and Dr. Hood of Fairview, was called in. He was an old practitioner, and when he came and had a history of the case, and examined for himself, he frankly told the patient and her friends that he had never seen or read or heard of a case of the kind. I stated to him what I had done, what I tried to do and failed, what I thought should be done if the patient would submit. He agreed with me as to the nature of the case, the impossibility of forcing the os uteri by manipulation and medication; but was of opinion that nature would throw off the child by decomposition, if we would use means to sustain our patient's strength. I thought otherwise, and insisted on dividing the ring and delivering. But in the absense of any authority or precedent to sustain me, I submitted to my senior's opinion, and left the case to nature, assisted by wine, porter, quinine and nourishing diet. The case progressed gradually, her general health gave way, and in the month of July following she died, one of the most heartrending objects I have ever witnessed.

The soft parts of the child all passed away by decomposition, leaving nothing but the dry bones, some of which I removed through the ring before death, but the larger ones could not pass.

He says: "By reporting this case I do not expect to add anything to what has already been written by others; but it is one more case, and in fact the first one in point of date of which he has any knowledge." This case lasted from February to July (five months.)

The next and fifth case occurred in my practice in July, 1863. Although the history of the case is somewhat unsatisfactory previous to my visit; yet in its result, and as regards treatment, will make but little difference. The case was that of an Irish woman, who was very ignorant, as was her husband and midwife. I first visited her July 20th, and from the midwife learned she had been in labor, as she supposed, a week; but the pains had gradually subsided, and for this strange phenomenon I was sent for. I found her weak and sallow, evidently suffering from some prostrating influence. I made an examination and found the os uteri open enough to introduce two fingers, but as hard and unyielding as an ivory ring, and inside the womb could readily feel the cranial bones, which were separated from each other by decomposition, the rough serrated edges almost cutting my fingers, evidently showing that the case had been one of some duration. The death of the child must have occurred some time before.

I tried to dilate the os by manipulating with my fingers, sufficiently, to get away the loose denuded bones, but was unsuccessful. I gave ergot but with no success—day after day I tried various remedies, but it seemed as though the os was totally incapable of dilatation. She was evidently sinking rapidly. I had never before seen or read of a similar case. I asked and obtained a consultation. Dr. Roach, a very excellent young physician, was called in, and found the case as above stated, and tried pretty much the same means that had already been employed, and wi h as little success. In consultation I gave as my opinion that the os uteri would never yield, that she was rapidly sinking, and from the absorption of the putrid gasses, she would soon die of pyemia if not relieved—and advised cutting the ring and making an opening large enough to relieve her of the putrid mass.

The Doctor thought it hazardous, and as the case was a new and singular one, thought that by tonic treatment nature might relieve her by throwing it off by decomposition. I consented and used the means indicated. She gradually sank into a typhoid condition, and died in four days after my first visit.

Cases of uterine abortion, like the foregoing, are necessarily rare, and as far as I can learn, always fatal. It seems when the uterine contraction does not take place, or subsides after having taken place at the full period of utero gestation, they can never be awakened again. Why this is so we cannot certainly say, but it seems to be dependent upon the death of the child, and the paralysing effect produced upon the nerves and muscular fibres of the womb by the putrifying child—which renders the normal expulsion of the child impossible.

Although the cases cited differ from the one that came under my observation in point of duration, yet the treatment would be the same. From the effect produced upon the mother, and the total separation of the bones of the head in this case, I am led to the conclusion that the child must have been dead some time. There was no secretion of milk, or if there was, I was not informed of it.

We now come to the all important subject, how are cases of "missed labor" to be treated? Dr. Williams, in the transactions of the Obstertrical Society of London, says, as soon as a physician's attention is called to a case of this kind, death of the child, escape of the liquor amnii, and os dilatable, to turn and deliver. In the cases stated, dilatation was impossible, and nothing but a mass of bones to turn. He then said no accoucheur would any more leave a dead fœtus than a putrid placenta in the womb. That he wonld advise dilatation by the tampon or incising the os. Dr. M. Green, in his case advocates dividing the ring and delivering the child, but as yet no case has been operated on, and as far as the cases have been recorded, not one has recovered, and it is to this dark record that I wish to call the attention of the profession, and influence them in trying other means of relieving the patient, than the ones heretofore tried. And that is, in incising the os uteri enough to relieve the woman of the putrid mass, conceiving as I do, that a clean incision, and ridding the womb of its poisonous load, would give us better hopes of recovery than to wait for nature to rid it of its contents; especially as in all recorded cases, death was the inevitable result, sooner or later, by the expectant treatment.

I am anxious to learn of any other cases that may have fallen under observation, and hope they will be reported.

